# Undergraduate medical education for neurodivergent students: a scoping review

**DOI:** 10.1186/s12909-025-08447-2

**Published:** 2025-12-13

**Authors:** Emily J. Green, Megan E.L. Brown, Gillian H.S. Vance, Iain D. Keenan

**Affiliations:** School of Medicine, Newcastle University, Framlington Place, Newcastle upon Tyne, NE2 4HH UK

**Keywords:** Neurodiversity, Undergraduate, Medical schools, Medical students, Teaching, Education

## Abstract

**Background:**

Recognition of the importance of the neurodiversity paradigm is growing within medical education, as is understanding of how current practices may create barriers for neurodivergent students. This review aims to explore existing empirical research regarding undergraduate medical education for neurodivergent students, in order to provide practical considerations for educators and inform planning of future research.

**Method:**

Following frameworks outlined by Arksey and O’Malley and the PRISMA Extension for Scoping Reviews, six electronic databases were searched in April 2024 for empirical studies relating to neurodiversity within undergraduate medical education. Quantitative synthesis of article characteristics and a thematic analysis of qualitative studies on student experience were conducted.

**Results:**

Fifteen (*n* = 15) studies were identified, relating to student experience, assessment, or staff perceptions. Most focussed on dyslexia or specific learning disabilities, with few explicitly referencing the neurodiversity paradigm. Studies of student experience frequently identified discrimination and stigma, and studies of staff perceptions highlighted inadequate training. Barriers to neurodivergent student education were linked to certain teaching modalities and learning environments. Several perceived strengths of neurodivergence were also noted. Studies on assessment focused on the role of accommodations, with an emphasis on written rather than clinical examinations.

**Conclusions:**

This review highlights a lack of empirical studies on neurodiversity within undergraduate medical education, restricting the development of pragmatic guidance. Some adaptations are suggested, but studies are limited to discussing medical education in general with few implementing the neurodiversity paradigm. Future research should explore a greater range of neurodivergent conditions, interrogate assessment practices including clinical examinations, and provide further evidence for inclusive teaching approaches. Explicit utilization of the neurodiversity paradigm is crucial, to amplify neurodivergent voices and better understand existing barriers. This work will have implications for medical educators seeking to understand neurodivergence, with a view to implementing adaptations for neurodivergent students in their educational practice.

**Supplementary Information:**

The online version contains supplementary material available at 10.1186/s12909-025-08447-2.

## Background

Increasing numbers of students in Higher Education Institutions (HEIs) are disclosing a specific learning difficulty (SpLD) and/or Autism [1–3]. This has coincided with greater recognition of the importance of the ‘*neurodiversity*’ paradigm. Within the paradigm, ‘*Neurodivergence’* is described as referring to *“those who differ from societally perceived norms of brain or mind function—or from the majority*,* who are sometimes described as ‘neurotypical’” *[4]. The implications of this paradigm for medical education have recently been outlined [4], including requiring neurodivergence to be understood as a social justice concept that demands critical reflection of traditional perspectives. Neurodivergent students in HEIs face several challenges, including greater attrition [5, 6], poorer mental health [7, 8], and less favourable post-graduation outcomes [9]. The demands of medical curricula [10–12] mean it is important to explore existing research regarding whether these broader challenges are prevalent in neurodivergent undergraduate medical students, including if they experience any specific difficulties, and what appropriate teaching and support strategies can be implemented. Improving our understanding in this area is even more crucial given recent political threats to diversity, equality and inclusivity initiatives [13]. Actively including neurodivergent students is vital to create equitable and just medical education [14], whilst developing a diverse workforce that can better support diverse patients [15–17].

### The neurodiversity paradigm

There is debate regarding the precise definition of *neurodiversity* [18, 19]. The definition described here, of the *neurodiversity paradigm*, draws on Shaw et al., (2024) [4] and other neurodivergent-authored texts [20], and aims to provide a clear explanation while also capturing the political essence of the paradigm.

Development of the *neurodiversity paradigm* involved many (often online) Autism advocate communities during the 1990–2000s [18, 21], as well as individuals such as Jim Sinclair [22], Harvey Blume [23] and Judy Singer [24]. Singer produced sociological studies on neurodiversity, but is often incorrectly credited with coining the term, and her views have been criticized by Autistic communities [21, 25]. The *neurodiversity paradigm* challenges traditional ‘deficit-driven’ models of Autism [20], that are associated with the medical model of disability, which views Autism as a ‘deficit’ to be ‘fixed’ or better aligned with society’s perception of ‘normal’ [18, 26]. Instead, the *neurodiversity paradigm* describes the variability in neurodevelopment [20, 27, 28]. This echoes the social model of disability [20] by arguing that difficulties faced are the result of societal barriers [26]. *Neurodiversity-aligned* or -*affirmative* research promotes improving quality of life, social justice [4], and including neurodivergent people within research designs [19, 29]. The *neurodiversity paradigm* is applied to various conditions [20], including ADHD (Attention-Deficit Hyperactivity Disorder), dyspraxia, and SpLDs, opposing pathologization and highlighting strengths [28, 30]. Within the paradigm, individuals may be described as ‘*neurodivergent’* if their neurocognitive function differs from societal ‘norms’. These differences in cognitive or neurological function set neurodivergence apart from personality traits or preferences. Populations are described as ‘*neurodiverse’* if they include individuals with a variety of neurocognitive ‘styles’ [20]. ‘*Neurodivergence’* is therefore considered a broad term for a variety of diagnoses, and ‘*neurodiversity’* acts as a paradigm for viewing research though a lens of social justice and inclusivity.

### The neurodiversity paradigm in medical education

Neurodivergence is present amongst medical students [31–37], but is likely higher than reported, due to underdiagnosis and under-disclosure [31]. Under-disclosure often results from stigma [38] or poor understanding from staff or peers [31, 39, 40]. Delayed diagnosis is common [33], as students can often compensate until medical school demands and/or assessment procedures exceed their compensatory capacities [41, 42]. Barriers and delays to diagnosis/disclosure are problematic, as universities often require disclosure of a formal diagnosis before providing the accommodations (such as examination adjustments) [43–46] that allow neurodivergent students to thrive [9] and showcase their strengths [7, 38].

Existing literature in medical education includes some discussion articles on neurodiversity [4, 38, 42, 47] and some reviews regarding SpLDs [48–52], but there are no existing reviews regarding neurodiversity within undergraduate medical education. A recent review by Gray et al., (2025) has scoped the literature regarding neurodivergence within the broader field of health professions education [53], which provides a helpful overview across many studies and disciplines. This indicates growing interest in this area and marks a useful starting point in understanding the evidence base for neurodiversity within health professions education. However, the wide scope of the Gray et al., (2025) review means only high-level, broad conclusions can be drawn, providing fewer practical implications for educators. Here, we focus on studies identified within the context of undergraduate medical education. Several factors set medical degrees apart from other healthcare courses. For example, medical degrees are ‘generalist’ [54], allowing graduates to specialize in any healthcare area, unlike dentistry, pharmacy, or midwifery, which focus on specific systems. There is typically a pre-clinical/clinical structure in medical programs [55]; a pre-clinical focus on foundational medical sciences is less prominent in wholly clinical-based programs, such as nursing [56]. These factors create a unique environment for medical learners. Furthermore, the Gray et al., (2025) review did not include detailed discussion of the definition of the neurodiversity paradigm, as we have sought to provide in our introduction. Therefore, our scoping review seeks to build on the work of Gray et al., (2025), by providing more in-depth discussions of studies that have considered neurodivergence specifically within the context of undergraduate medical education. We focus on empirical studies, with a view to providing pragmatic conclusions that can guide medical educators in their daily practice. We have also sought to use this introduction to provide meaningful discussion of the neurodiversity paradigm, which is often lacking in existing studies on neurodivergence in medical education, which often give brief definitions that do not explore the nuance or sociopolitical aspects of the term. Given the growing recognition of the importance of the neurodiversity paradigm in medical education, and the diverse needs of this student population, a scoping review is required to provide an overview of existing evidence on this topic, including methodologies, key findings, and identifying where further research is required.

### Definitions and language in this review

The *neurodiversity paradigm* encompasses the variation in human minds, which drives social justice, equality and inclusion for those who fall outside of society’s definition of ‘normal’ - who may be described as *neurodivergent* (compared to the majority, who may be termed *neurotypical)*. Populations may be described as *neurodiverse*, capturing this variation across a group.

There is debate regarding person-first versus disability-first language [57], making it important to check for individual preferences. Here, we intentionally move between person-first and disability-first language, to attempt to include all viewpoints. Additionally, the word ‘difference’ may better align with the neurodiversity paradigm than ‘deficit’ or ‘disorder’ [58]. We use accepted medical abbreviations (‘SpLDs’, ‘ADHD’), but we acknowledge that other terms may be preferred. The authors consulted neurodivergent colleagues and neurodivergent-authored texts when developing definitions and terminology.

### Identification of research question

Our aim was to explore existing empirical research regarding undergraduate medical education for neurodivergent students, including determining which aspects of educational practice (e.g. assessment, student experience) have been studied in this context, identifying key findings and methodologies, in order to provide practical considerations for educators in their day-to-day practice and to identify possible evidence gaps to inform planning of future research. This is an appropriate rationale for carrying out a scoping review [59, 60].

The research question for this review was:

- What is the nature and extent of empirical research available to inform medical education approaches for undergraduate students who are neurodivergent?

## Methods

### Author reflexivity

The authors of this review are academics and educators, which may influence interpretation of data regarding student experiences. The authors have many years of experience in teaching and supporting medical students but have recently realized their own lack of knowledge regarding neurodiversity, what effect this can have on students, and how best to support them. The first author (EJG) is currently undertaking a PhD exploring the lived experience of neurodivergent students in anatomy education, and this scoping review has been carried out as an initial phase of this project.

The authors who carried out this review are neurotypical, which may influence interpretations of included studies, particularly for thematic analysis of qualitative studies. Review of neurodivergent-authored literature concerning the definition of the neurodiversity paradigm, and the experiences of neurodivergent individuals, was carried out prior to commencing this work, encouraging the authors to challenge any pre-existing ideas or assumptions held about neurodiversity. Reflection and discussion between the authors occurred throughout thematic analysis of qualitative studies, to ensure emergent themes reflected neurodivergent voices. Furthermore, on completion of the review process, one of the authors, who is neurodivergent, joined the project in order to provide a neurodivergent viewpoint, by reviewing and refining identified themes. Their contribution supported a more authentic interpretation of the findings and helped to challenge any unconscious biases or assumptions. This author gave further insight throughout the process of compiling the results, discussion and background of the scoping review. Many of the studies included in the review were authored by neurotypical individuals, adding further layers of interpretation that may shape how neurodivergent experiences are represented.

### Protocol

The scoping review protocol followed frameworks by Arksey and O’Malley [56], as updated by Levac et al. [60], and the PRISMA Extension for Scoping Reviews (PRISMA-ScR) [61]. The protocol was registered with the Open Science Framework (OSF): 10.17605/OSF.IO/4GSYN. A completed PRISMA-ScR checklist is available as Additional File 1.

This scoping review forms part of a wider review project regarding medical sciences and healthcare professions education, as part of the PhD work of the first author.

### Identification of relevant studies

Neurodiversity is a relatively novel concept within medical education; a brief search of existing literature prior to developing the protocol found few studies using this terminology. Although we use a broad definition of neurodiversity that does not apply to a specific list of conditions, it is necessary to select specific terms to be used in a search strategy. Therefore, as well as searching for studies including the word ‘neurodiversity’ or related terms, we also scoped the literature for studies on commonly recognized neurodivergent conditions, including Autism, ADHD, dyspraxia, and SpLDs. We acknowledge that there may be other conditions that could be considered under the neurodiversity paradigm, but our search strategy was limited to these conditions in order to return manageable search results. Studies on the prevalence of neurodivergence and on accommodations (e.g. studies regarding types of accommodation offered and how often) were excluded, as both prevalence and the securing of accommodations fall outside the direct influence of educators (accommodations are usually arranged by centralized university services). Studies regarding provision of accommodations are therefore more relevant to policymakers and administrators who make these decisions, rather than the individual educator. This review focuses on synthesizing available empirical evidence to inform day-to-day teaching practice and research priorities related to this. The research question informed the development of the inclusion criteria and search strategy. An example search strategy is available in Table 1, exact strategies are available in Additional File 2.

**Table 1:** Development of inclusion criteria and search strategy, informed by the research question. The search terms given are an example, searches were adapted as appropriate for each database with the support of an information specialist. Full search strategies for each database are available as additional file 2. Search strings for ‘medical education/students’ and ‘neurodiversity’ were joined by an ‘AND’ operator. An additional line, ‘NOT child*’ was added to the search, as initially a very high number of results regarding childhood neurodivergence were returned. ‘NOT’ operators must be used with caution to avoid excluding potentially relevant articles, this is discussed further in the limitations section

Question Element	Inclusion Criteria	Example Search Terms
*Medical*	Any healthcare professionals education (Medicine; Nursing; Dentistry; Pharmacy; Speech & Language Therapy) and/or medical sciences education (Biomedical Science; Anatomy; Physiology; Sport and Exercise Science; Nutrition). *Only studies identified relating to medicine and dentistry are discussed in this article.*	((medical OR dental OR nursing OR pharmacy) N6 school) OR ((medical OR dental OR nursing OR pharmacy) N6 education) OR ((medical OR dental OR nursing OR pharmacy) N6 student*) OR ((medical OR dental OR nursing OR pharmacy) N6 teach*) OR ((medical OR dental OR nursing OR pharmacy) N6 program*) OR ((medical OR dental OR nursing OR pharmacy) N6 curricul*) OR ((medical OR dental OR nursing OR pharmacy) N6 assess*) OR ((medical OR dental OR nursing OR pharmacy) N6 exam*) OR ((medical OR dental OR nursing OR pharmacy) N6 train*) OR ((medical OR dental OR nursing OR pharmacy) N6 study*) OR (anatomy N6 (education OR student* OR teach* OR program* OR curricul* OR assess* OR exam* OR train* OR study*)) OR (physiology N6 (education OR student* OR teach* OR program* OR curricul* OR assess* OR exam* OR train* OR study*)) OR (biomed* N6 (education OR student* OR teach* OR program* OR curricul* OR assess* OR exam* OR train* OR study*)) OR (sport* N6 (education OR student* OR teach* OR program* OR curricul* OR assess* OR exam* OR train* OR study*)) OR (nutrition N6 (education OR student* OR teach* OR program* OR curricul* OR assess* OR exam* OR train* OR study*)) OR (speech N6 (education OR student* OR teach* OR program* OR curricul* OR assess* OR exam* OR train* OR study*)) OR (“healthcare professional” N6 (education OR student* OR teach* OR program* OR curricul* OR assess* OR exam* OR train* OR study*)) OR (medicine N6 (education OR student* OR teach* OR program* OR curricul* OR assess* OR exam* OR train* OR study*)) OR (physician N6 (education OR student* OR teach* OR program* OR curricul* OR assess* OR exam* OR train* OR study*))
*Education or Students*	Undergraduate (HE) only. Empirical research only. Teaching/learning, assessment, student experience, student support, accommodations/adjustments/accessibility	
		*AND*
*Neurodivergent or Specific Learning Difficulty*	Any neurodivergence, for example (but not limited to) Autism, dyslexia, ADHD, dyspraxia, dyscalculia	neurodiver* OR autis* OR adhd OR “attention deficit” OR “learning disab*” OR “Intellectual disab*” OR “learning difficult*” OR “learning disorder*” OR “learning disturbance*” OR “learning impairment” OR “learning problem*” OR “attention deficit*” OR “attention disturbance*” OR dyslexi* OR dyspraxi* OR dyscalcu* OR aphantasi* OR “autis* spectrum”
		NOT child*

The search was not limited by date, journal, language or study type. Articles that were excluded due to study type or language were removed later in the process during the screening phase. The strategy was created with an information specialist, using both subject headings and keywords (Table 1). The search was conducted on 19 April 2024 in six electronic databases: Medline, Embase, PsycInfo, ERIC, Social Sciences Premium Collection and Web of Science, supplemented with a gray literature search using Google Search (first page of results only) and Google Scholar (first three pages of results). Backwards citation searching of included studies did not identify further articles. We repeated the search on 04 August 2025 with no further articles identified.

### Study selection

Citations were exported to EndNote (EndNote Version 20, Clarivate, Philadelphia, PA) and then screened using Rayyan (Rayyan, Cambridge, MA). Duplicates were identified and removed prior to study selection, which was performed in three steps: title and abstract screening, and full-text screening, according to the exclusion criteria. Studies were excluded if they:

- Did not relate to education or students, e.g., were related to staff who are neurodivergent/have SpLDs. Studies regarding prevalence of conditions amongst medical students were excluded. Studies relating to frequency/type of accommodations offered were excluded (e.g. studies regarding types of accommodation offered and how often) as accommodations are usually managed by centralized university services and therefore not under the direct influence of educators
- Did not relate to medical students
- Did not relate specifically to neurodivergent students
- Were related to admissions/applications to study
- Were related to qualified health professionals or postgraduate, rather than undergraduate students
- Were not empirical research/original study (e.g. opinion, commentary, literature review)
- Were not available in English

For pragmatic reasons (including time available and researcher capacity), title and abstract screening was performed by a single researcher (EJG), who took a cautious approach, progressing studies to full-text screening if there was any doubt regarding their inclusion. Full-text screening was carried out independently by two researchers (EJG, IDK) to ensure consistent adherence to the exclusion criteria. Conflicts were resolved through discussion and consensus; most discussion took place around inclusion or exclusion of studies related to accommodations or prevalence with the ultimate decision to exclude these, as discussed previously.

### Extracting and charting the data

We developed a data extraction tool (available as Additional File 3) using Microsoft Excel to collate key information from included studies, determined by the research question and aims, including: article metadata; methodology; participants; author characteristics; key objectives and results. Extraction categories were determined through familiarity with the literature and discussion amongst the screening team (EJG, IDK). Data extraction for all studies was carried out by a single researcher (EJG); a second researcher (IDK) extracted the data for a random sample of 50% of included articles, with conflicts resolved through discussion and consensus.

### Collating, summarizing and reporting results

We developed tables and charts to visualize characteristics of the included studies, consisting of quantitative synthesis of article meta-data and characteristics, and a thematic analysis of qualitative studies on student experience. Qualitative studies on student experiences underwent inductive thematic analysis [62], involving coding of these studies to identify common results through repeated reading and familiarization of included articles by a single researcher (EJG). Codes were then organized into emergent themes, that captured and summarized key findings across the articles. Themes were discussed and refined with a second researcher (IDK). Quality evaluation was not performed as this is not a requirement of scoping reviews [59, 60].

## Results

### Search results

**Fig. 1:**
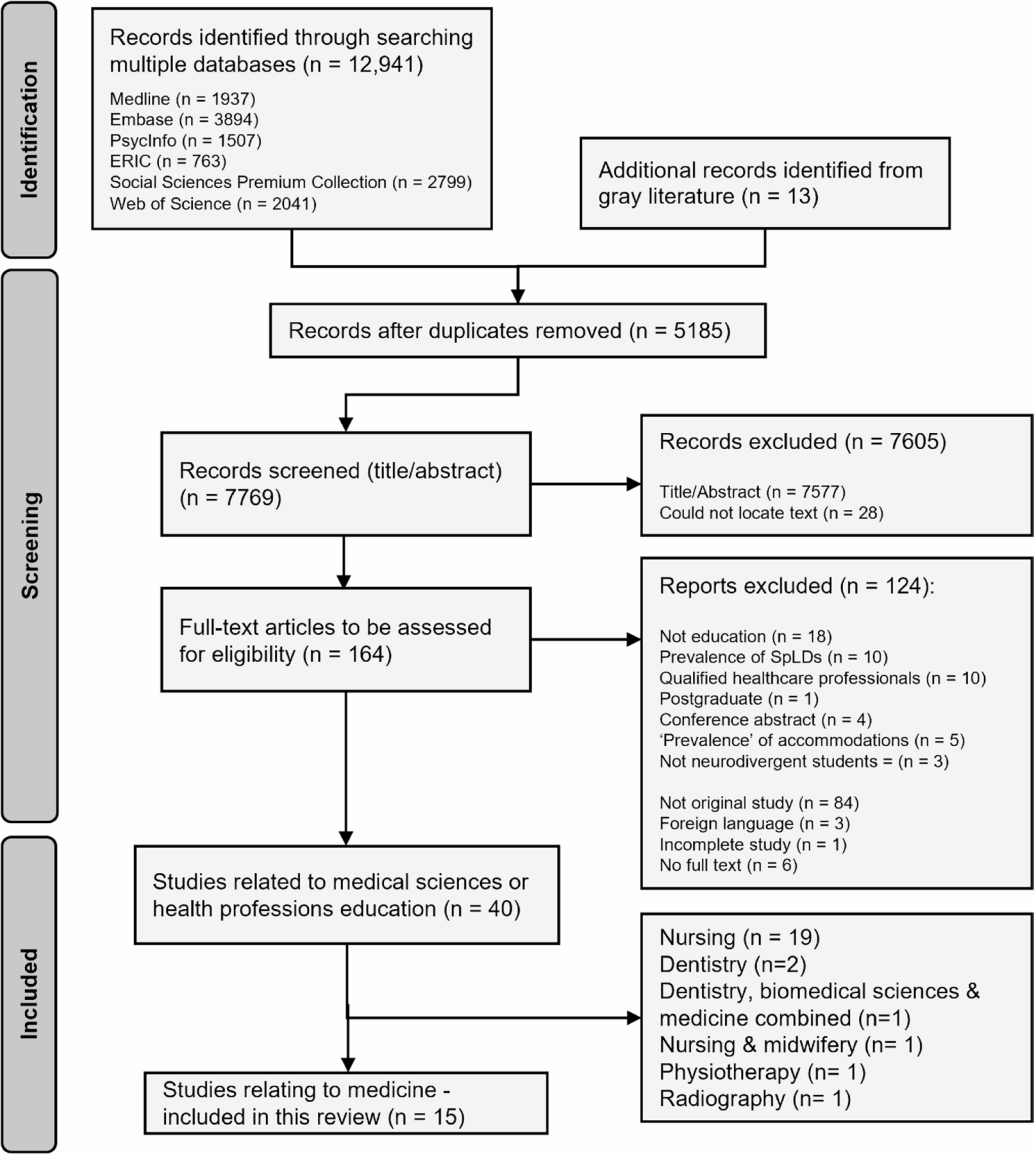
A preferred reporting items for systematic reviews and meta-analyses extension for scoping reviews (PRISMA-SCR) [61] flowchart of the literature selection. Some articles met more than one of the exclusion criteria, meaning the listed reasons for exclusion totals more than the overall number of excluded articles. Four conference abstracts were excluded on the basis of one being an incomplete study, one concerning prevalence of neurodivergence (education was not the main focus), and two were not original studies (literature review)

The search results are outlined in a PRISMA-ScR [61] flowchart (Fig. 1). A total of 12,941 publications (*n* = 12,941) were identified through database searching and 13 through grey literature searching (*n* = 13).

After removing duplicates (*n* = 5185), we screened the remaining publications (*n* = 7769) according to exclusion criteria (Fig. 1). Fifteen (*n* = 15) included studies related to medicine [63–77]. A summary of included studies is shown in Table 2, with full details listed in Additional File 4. Our focus on undergraduate medicine and exclusion of studies related to accommodations or prevalence resulted in fewer included studies regarding medical education than in Gray et al., (2025) [53].

**Table 2:** Table summarizing the key details of the 15 included studies. The table shows the nature of the condition focused on by each study, the study design and location, whether participants were staff or students, and the total number of participants

Included Study	Location	Condition Studied	Study Design	Nature of participants (staff or students)	Number of participants
Anderson JL, Shaw SCK. The Experiences of Medical Students and Junior Doctors with Dyslexia: A Survey Study. International Journal of Social Sciences & Educational Studies. 2020−11−26 2020;7(1):62–71.	UK	Dyslexia	Survey	Students (Junior doctors discussing their experiences as students)	77
Bailey A, Grotowski M, Bailey S. Medical education: Accommodating students with ADHD. Medical teacher. 2023:1–6.	Australia	ADHD	Survey pilot	Staff	16
Gibson S, Leinster S. How do students with dyslexia perform in extended matching questions, short answer questions and observed structured clinical examinations? Advances in health sciences education : theory and practice. 2011;16(3):395–404.	UK	Dyslexia	Statistical analysis of examination data	Students	686 with no declared dyslexia, 91 with dyslexia
Godfrey-Harris M, Shaw SCK. The experiences of medical students with ADHD: A phenomenological study. PloS one. 2023;18(8):e0290513.	UK	ADHD	Interpretive phenomenological analysis of semi-structured interviews	Students	6
Gray CP, Burr SA. Timing is key to providing modified assessments for students with specific learning difficulties. Perspectives on medical education. 2020;9(1):49–56.	UK	SpLDs	Statistical analysis of examination data	Students	108
Hennessy LR, Shaw SCK, Anderson JL. Medical Students’ Attitudes towards and Beliefs about Dyslexia: A Single-Centre Survey Study. International Journal of Social Sciences & Educational Studies. 2020; 7(4):69–79.	UK	Dyslexia	Survey	Students	123
Magnin E, Ryff I, Moulin T. Medical teachers’ opinions about students with neurodevelopmental disorders and their management. BMC medical education. 2021;21(1):16.	France	Neuro-developmental disorders	Survey	Staff	67
McKendree J, Snowling MJ. Examination results of medical students with dyslexia. Medical Education. 2011;45(2):176–182.	UK	Dyslexia	Statistical analysis of examination data	Students	544 (36 with dyslexia)
Ricketts C, Brice J, Coombes L. Are multiple choice tests fair to medical students with specific learning disabilities? Advances in health sciences education : theory and practice. 2010;15(2):265 − 75.	UK	SpLDs	Statistical analysis of examination data	Students	763 (50 with SpLD/dyslexia)
Rowlands A, Abbott S, Bevere G, Roberts CM. Medical students’ perceptions and understanding of their specific learning difficulties. International Journal of Medical Education. 2013; 4:200–206.	UK	SpLDs	Framework analysis of semi-structured interviews	Students	15
Shaw SCK, Anderson JL, Grant AJ. Studying Medicine with Dyslexia: A Collaborative Autoethnography. The Qualitative Report. 2016;21(11):2036–2054.	UK	Dyslexia	Interpretive phenomenological analysis of unstructured interviews	Students (Junior doctors discussing their experiences as students)	8
Shaw SCK, Anderson JL. The experiences of medical students with dyslexia: An interpretive phenomenological study. Dyslexia (Chichester, England). 2018;24(3):220–233.	UK	Dyslexia	Collaborative autoethnography, including an autobiographical account and interview	Student	1
Shaw SCK, Doherty M, Anderson JL. The experiences of autistic medical students: A phenomenological study. Medical education. 2023;57(10):971–979.	UK	Autism	Interpretive phenomenological analysis of semi-structured interviews	Students	5
Shaw SCK, Hennessy LR, Anderson JL. The learning experiences of dyslexic medical students during the COVID-19 pandemic: a phenomenological study. Advances in health sciences education : theory and practice. 2022;27(1):107–124.	UK	Dyslexia	Interpretive phenomenological analysis of semi-structured interviews	Students	5
Walker ER, Shaw SCK, Anderson JL. Dyspraxia in Medical Education: A Collaborative Autoethnography. The Qualitative Report. 2020;25(11):4072–4093.	UK	Dyspraxia	Collaborative autoethnography, including an autobiographical account and interview	Student	1

### Study characteristics

**Fig. 2:**
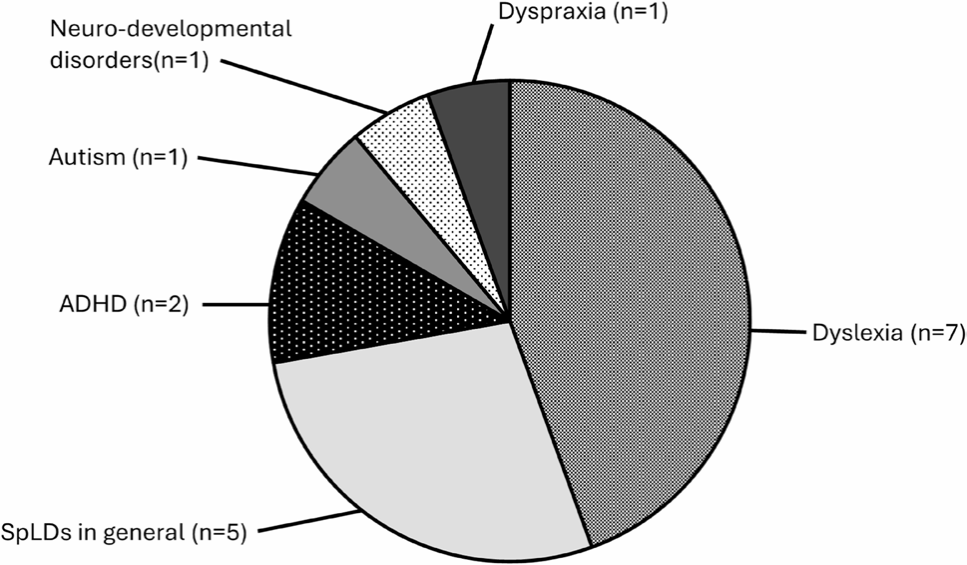
Chart showing the distribution of SpLDs or neurodiverse conditions discussed by the included articles. Most articles discussed SpLDs (*n* = 14), with the majority of these discussing dyslexia (*n* = 7) or SpLDs in general (*n* = 5)

The fifteen included studies were published between 2010 and 2023, taking place in the UK (*n* = 13) [63, 65–68, 70–77], Australia (*n* = 1) [64], and France (*n* = 1) [69]. Eight (*n* = 8) were single-centre studies [64–68, 70–72, 76], two were collaborative autoethnographies (*n* = 2) [74, 77], one involved staff members from two universities (*n* = 1) [69], and three involved participants from across the UK (*n* = 3) [63, 73, 75]. Most discuss dyslexia (*n* = 7) [63, 65, 68, 70, 73, 74, 76] or SpLDs in general (*n* = 3) [67, 71, 72]. Fewer discussed ADHD (*n* = 2) [64, 66], Autism (*n* = 1) [75], dyspraxia (*n* = 1) [77] or neurodevelopmental disorders (*n* = 1) [69] (Fig. 2). Only four (*n* = 4) studies referenced ‘neurodiversity’ (or related terms) [64, 66, 75, 76]. Of these, two described ADHD as an example of neurodivergence without defining neurodiversity [64, 66], one briefly discussed neurodiversity as a theoretical lens similar to the social model of disability [76], and one gave a more expansive definition [75].

Eight studies (*n* = 8) disclosed that an author was neurodivergent [63, 64, 66, 73–77], with four (*n* = 4) of these including contributions from a student author [64, 66, 74, 77]. Seven of the eight studies that disclosed the neurodivergence of their authors included the same author (S. Shaw) as a contributor (*n* = 7) [63, 66, 73–77].

### Study participants and aims

The majority of studies involved student participants (*n* = 13 studies). Two studies had staff participants (*n* = 2) [64, 69], including healthcare professionals involved in teaching [64] and medical teachers [69]. Of the studies involving student participants, these were mainly neurodivergent undergraduates (*n* = 10 studies) [65–67, 70–72, 74–77], although two studies involved junior doctors discussing their student experiences (*n* = 2 studies) [63, 73] and one study had neurotypical student participants (*n* = 1 studies), examining their perceptions of dyslexia [68]. Eight studies required participants to be formally diagnosed or registered with university support services in order to participate (*n* = 8) [65, 67, 70–73, 75, 76]. Other studies did not require this (*n* = 1) [66], did not state whether this was required (*n* = 1) [63], or this was irrelevant as the participant was an already diagnosed author (*n* = 2) [74, 77].

Included studies explored one of three areas: student experiences/perceptions [63, 66, 68, 72–77]; assessment/academic performance [65, 67, 70, 71]; or staff perceptions [64, 69].

### Student experiences and perceptions

Eight studies investigated the experiences/perceptions of neurodivergent students (*n* = 8) mostly utilizing semi-structured interviews [66, 72, 73, 75, 76]. Two studies (on dyslexia and dyspraxia) used autoethnography of a single participant [74, 77] (*n* = 2). Two used mixed-methods surveys (*n* = 2), one to investigate experiences of students with dyslexia [63], and one to investigate the views of students without SpLDs towards dyslexia [66].

Thematic analysis of studies with neurodivergent participants identified common experiences, regardless of condition (Table 3). Themes included: diagnosis, disclosure and discrimination; teaching, learning environment and adjustments; mixed clinical experiences and communication; emotional impact, strengths and support.

**Table 3:** Themes identified through thematic analysis of qualitative studies on neurodivergent student experiences and perceptions. These themes discuss experiences that were seen across studies on different diagnoses, however it is important to note that although many experiences appear common, experiences across diagnoses are not homogenous. Practice points or areas where further evidence is required, that medical educators and/or researchers may wish to consider, have also been summarised

Theme	Description
1. Diagnosis, disclosure & discrimination	Students noted the importance of early diagnosis and disclosure [67, 73, 75]. Barriers to this included poor reactions or inadequate understanding from staff [64, 67, 74–76], experience of discrimination or stigma [67, 74–76], or bullying from staff [64, 67, 74] or from peers [64, 74, 76, 77]. **Practice points**: Medical educators should take steps to learn more about neurodivergent conditions in order to better understand the impact of these on students; medical schools may also wish to consider developing staff training on this. This learning should be carried through into curricula to foster an environment of respect and challenge stigma amongst fellow students. Understanding how best to integrate teaching around neurodiversity into curricula would be a valuable avenue for future studies.
2. Teaching, learning environment & adjustments	Lectures [64, 67, 76, 77], transitions (from school, from pre-clinical to clinical years, and between placements) [64, 67, 77], and timetabling changes or poor structure [67, 76, 77] were all notable barriers. Several studies reported neurodivergent students needing to work harder than their peers for the same outcomes, including efforts in double-checking or over-preparing [64, 74, 75, 77]. Adjustments were appreciated [64, 75, 76] but some described these as performative and suggested they could be better individualized and tailored to the medical degree, as accommodations were generally provided via central university services rather than the medical school [67, 75, 76]. **Practice points**: Consistent timetabling and efforts to minimize changes is helpful for neurodivergent students. Lecture theatre environments are potentially prohibitive; further research into more appropriate learning spaces or approaches would be useful to guide educators on effective alternatives.
3. Mixed clinical experiences & communication	Four studies described clinical placement learning as important, identifying pre-clinical teaching as more challenging for neurodivergent students [75–77]. Three studies discussed supportive experiences with clinical staff [67, 75, 77], but three reported difficulties caused by poor awareness of clinical placement staff about students’ neurodivergence [67, 75, 76]. Three studies found neurodivergent students felt able to communicate well with patients, aided by defined social roles and their strong sense of empathy [74–76], whereas communication with neurotypical peers or staff is more difficult [67, 76, 77]. **Practice points**: The pre-clinical years may be a particularly challenging time for neurodivergent students, and medical schools may wish to consider additional support for students at this stage, allowing them to showcase strengths in the clinical environment. Improved processes for sharing information about students’ neurodivergence with staff on clinical placements reduces the need for forced disclosure or difficult situations arising from lack of awareness from clinical staff; sharing of best practice if any institutions have achieved this would be beneficial.
4. Emotional impact, strengths & support	Difficulties were compounded by ‘toxic competitiveness’ at medical school [67, 74, 76, 77], and feelings of helplessness or hopelessness when attempting to seek support [67, 74–76]. Students identified feelings of stupidity, inadequacy and low self-esteem, resulting in perfectionism [64, 67, 74, 75, 77] and impacts on mental health [64, 67, 75]. In five studies, students described how their experiences as a neurodivergent student had affected their career choices or aspirations [64, 67, 73, 74, 77]. Students perceived strengths arising from their neurodivergence, including developing alternative approaches to learning [67, 73–75], perseverance/resilience [74, 76, 77], lateral thinking [67, 74, 75] and enhanced empathy [67, 75, 76]. Students in four studies mentioned a desire for better awareness of fellow neurodivergent students (e.g. via support groups), or role-modelling from neurodivergent educators or doctors [64, 67, 74, 76]. **Practice points**: Support groups and/or role modelling from neurodivergent doctors is considered helpful for neurodivergent students, which may also be a useful approach for addressing the mental health impacts of being neurodivergent. Research regarding wider medical school culture, how this impacts on neurodivergent students and other minority groups, and how medical schools can address this, is an important area for further work.

These themes were seen across studies on varying conditions, but some experiences appeared to be unique to particular diagnoses. It is important to note that although many experiences appear common, experiences across diagnoses are not homogenous, and being too quick to generalize may erode this nuance. For example, the study on autism noted strengths such as attention to detail and organizational skills, but difficulties or differences in sensory processing which created challenges in some learning environments, that were not mentioned in studies on other conditions [75]. Challenges in group work, as a result of managing social expectations and masking, were also identified in students with Autism [75]. Masking occurs when societal pressures result in neurodivergent individuals feeling as though they need to hide or reduce their neurodivergent traits, in order to be better accepted socially in environments that reward neurotypical norms [78, 79]. The high energy expenditure associated with masking was identified in studies of both Autistic students and those with ADHD, resulting in fatigue and therefore impact on learning [66, 75]. Despite this, students with ADHD expressed preference for small group learning compared to lectures [66]. Studies on dyslexia discussed challenges caused by assessments (such as OSCEs (Objective Structured Clinical Examinations)) not accurately reflecting the real clinical environment, and therefore not enabling students to showcase their strengths in clinical practice [73, 74]. The collaborative autoethnography regarding dyspraxia described physical difficulties with practical skills [77]. There were five studies focusing on experiences of students with dyslexia (*n* = 5) [63, 68, 73, 74, 76] but only a single study on SpLDs in general [72], ADHD [66], Autism [75] and dyspraxia [77]. The small number of included studies means further work is required to elucidate whether these examples are diagnosis-specific or common across neurodivergences, particularly as experiences can vary even within the same diagnosis [66, 73, 74].

The study on neurotypical student perceptions of dyslexia [68] found the majority of students had a good understanding of dyslexia and how it can affect dyslexic students, and supported adjustment for their dyslexic peers. A small number noted feelings of jealousy or that adjustments gave an unfair advantage, which may underpin the experiences of stigma often associated with neurodivergent students avoiding disclosure or seeking a diagnosis [38].

### Assessment approaches and academic performance

Four single-centre studies investigated assessment or academic performance for students with SpLDs [67, 71] or dyslexia [65, 70]. There were no studies regarding assessment for students with Autism, ADHD, or dyspraxia. Of the four studies, three carried out statistical analysis of examination results across multiple assessment types, in order to compare these between students with and without dyslexia [65, 70] or SpLDs [71]. Two additionally compared results for neurodivergent students with and without additional time as an accommodation [65, 71]. The fourth study compared examination performance between students with SpLDs who had accommodations, and students without accommodations [67]. Additionally, students who were diagnosed with an SpLD and provided with accommodations partway through the programme allowed comparison of performance pre- and post-accommodations being put in place.

SpLDs appear to have no significant effect on performance in multiple choice tests [71], when students with SpLDs are given exam adjustments, such as additional time. Extra time has been reported as significantly enhancing exam performance for students with SpLDs (P = < 0.05) [67] when progress-test performance (tests taking place four times per year) is compared pre- and post- receiving this adjustment, although it was found it can take up to a year for improvements to reach significance (for example, students who were diagnosed and received extra time during second year showed significantly improved performance in third year assessments, compared with those diagnosed and given extra time within third year (P = < 0.05)) [67]. Another study found students with dyslexia across two cohorts were found to perform significantly worse than non-dyslexic peers in year one (P = < 0.05 and < 0.01) [65]. No significant difference was seen in later years. They also found that dyslexic students given extra time in written assessments performed significantly better in year one than those not yet receiving this due to lack of diagnosis or disclosure (P = < 0.05) [65], emphasizing the importance of early adjustments.

There were differing results regarding OSCEs for students with dyslexia. One study found dyslexic students in first year performed significantly worse in OSCE examinations across three cohorts (P = < 0.05), and when combining these cohorts found significant differences in performance on certain stations (P = < 0.01) [65]. However, another study found no significant effect of dyslexia on examination performance across all assessment types, including OSCE, when comparing results between students with and without dyslexia [70].

### Staff perceptions of teaching and supporting neurodivergent students

Two studies used surveys to investigate staff perceptions of teaching students with ADHD [64] (*n* = 2) or neurodevelopmental disorders [69]. Both identified limited understanding about these conditions and identified a desire for further training.

## Discussion

To identify key themes and gaps in the existing literature on undergraduate medical education for neurodivergent students, this review has discussed 15 relevant studies. These studies investigated student experiences, staff perceptions, and assessment. We initially discuss studies on assessment; these positivist works quantify the impact on assessment for students who are neurodivergent, however these studies are not able to capture their broader lived experience which extends beyond that of assessment and academic achievement. We therefore then discuss the included interpretivist qualitative studies, which, when combined with positivist data, provide a more well-rounded view that can illustrate the experiences of these students and provide implications for practice.

### Accommodations can reduce barriers in written assessment, but work is needed to further investigate assessment practices themselves

Three single-centre studies found no significant difference in written exam performance between students with dyslexia or SpLDs and those without, when accommodations are provided [65, 70, 71]. The role and power of assessment accommodations in supporting neurodivergent students is important, as it has been widely recognized that differential attainment is a challenge faced by disabled medical students, including those with cognitive or learning disabilities [3, 80]. The studies in this review indicate that proper provision of accommodations may be a crucial step towards remediation. Accommodations are generally managed and provided by central university services, so medical schools should work closely with these departments to ensure correct procedures are in place to allow medical students to access the adjustments they need.

The included studies regarding assessment did not compare different SpLDs, and this review found no studies regarding assessment for students with Autism, ADHD or dyspraxia, meaning we cannot determine if different diagnoses affect assessment performance and the role of accommodations differently. This is an important area for future work. Additionally, assessment practices vary between medical degrees [81], meaning studies performed within the context of individual institutions are required.

Two studies found differing results regarding OSCE performance [65, 70], demonstrating a need for further research, particularly as accommodations are less frequently offered for clinical assessments [70]. Neurodivergent students report that lack of adjustments for clinical assessments results in anxiety, in turn causing students to rush and make mistakes [82], however some educators argue against accommodations as performing clinical tasks under pressure is essential for practicing clinicians [70]. Such arguments speak to the wider structural and cultural barriers facing neurodivergent medical students. Previous work regarding the experiences of disabled students has identified a ‘capability imperative’ within medical education [83], whereby a historic culture of ‘*compulsory hyper-ablebodiedness and mindedness’* has created assessment standards that may themselves be inherently ableist. A simplistic view of providing accommodations to allow neurodivergent students to meet existing standards or perform under existing assessment approaches does not acknowledge the systemic exclusion that underpins these practices. There is important work to be done to support medical schools in dismantling these cultures, as none of the studies regarding assessment identified in this review sought to consider the inclusivity of assessment practices themselves, making this an important future area to explore.

### Neurodivergent students face barriers in medical education beyond assessment, and there is a literature gap regarding appropriate teaching modalities

Studies on neurodivergent student experiences highlight challenges beyond assessment. Barriers to disclosure, including stigma, low self-esteem, poor reactions from staff or lack of understanding from peers are common [66, 73–75, 84] and seen amongst other healthcare students [82, 85–88]. Educators often lack understanding of neurodivergence [64, 69, 89, 90], and some express concerns about non-disclosure as a patient safety issue [91] (despite limited evidence [92]), or fear neurodivergent students may struggle to communicate with patients [89]. However, neurodivergent students are often hyper-vigilant, double-checking and taking extra time for clinical tasks [63, 73, 74, 77, 85, 93], and Autistic doctors report finding patient interactions straightforward, due to clear behavioural expectations and guidelines [43, 73–75]. Neurodivergent students suggest interpersonal relations with teachers and peers are more challenging [66, 75, 77]. Some argue this results from ‘weaponized professionalism’ [66], whereby those deviating from the construct of an ‘ideal’ medical professional (grounded in ‘*white*,* cis-gendered*,* heteronormative*,* able-bodied* [and neurotypical] *male experience’*) are viewed as unprofessional, regardless of whether their behaviour actually affects their ability to practice medicine [66, 94]. Medical schools and their governing bodies should ensure adequate training is provided for academic staff on neurodivergence, utilizing the neurodiversity paradigm to challenge cultural norms around what constitutes a ‘professional’ medical student, and to ensure educators appreciate the strengths and benefits neurodivergent students bring to the medical profession and community. Providing opportunities to elevate neurodivergent voices, such as through student support groups and role modelling from neurodivergent doctors, can also be an empowering approach.

Current teaching approaches, such as lectures, present barriers for neurodivergent students [66, 74–76]. Drawbacks of lectures are known [95], but these may be more significant for students who are neurodivergent. This review did not identify any empirical research investigating the most appropriate teaching modalities for neurodivergent students (existing articles are limited to opinion/commentary [50, 96, 97] or generic guidance [48]), nor any exploring specific curriculum elements (included studies only investigated medical education in general). Many neurodivergent students utilize alternative learning approaches [66, 72–74] and therefore appreciated flexible teaching approaches adopted during the COVID-19 pandemic (such as online or pre-recorded sessions) [76]. Further research regarding teaching approaches that can best support this student group is vital in order to guide educators in improving the inclusivity of their practice.

Commonly, barriers identified were known issues for many learners but were identified as more likely to be important for neurodivergent students [98]. Competitive medical school cultures [99], coupled with the ‘*invincibility myth’*, of doctors as being ‘superhuman’ and discouraged from disclosing difficulties [100], are known challenges for all medical students. These may exacerbate difficulties faced by neurodivergent students, by discouraging disclosure of what may be perceived as ‘weaknesses’, leading to perfectionism and over-compensation [66, 73, 74, 77]. None of the included studies explicitly investigated the potential intersectionality between neurodivergent student experiences and that of other minoritized groups [101, 102], an area certainly in need of further work within medical education. Supportive strategies that are likely to benefit all students are therefore crucial, an idea underpinning Universal Design for Learning (UDL) [103], which involves teaching being delivered using approaches that are accessible to all students and can therefore benefit a wider population. UDL is a popular concept [104–106], however recent work has identified a lack of evidence to support its use [107]. This lack of evidence means it is difficult to recommend UDL as a potential solution, until more robust evidence underpinning this approach can be identified. Changes may be needed on a systemic and policy level, but many educators would benefit from more detailed, pragmatic and actionable guidance on how to adapt their day-to-day teaching, which could benefit all students [108].

### Existing literature focuses on a small number of conditions with limited application of the neurodiversity paradigm

Most included studies concerned dyslexia or SpLDs in general; there is limited empirical research regarding undergraduate medical education for other SpLDs, ADHD, or Autism. This review identified many common experiences across diagnoses, which could simplify the development of supportive teaching strategies, but lack of studies on conditions beyond dyslexia and SpLDs weaken this conclusion. Compounding this gap is the lack of studies employing the neurodiversity paradigm. Most studies focus on a single condition, and this single-condition focus restricts opportunities to identify shared experiences across neurodivergences, hindering the development of educational interventions with wider applicability. The neurodiversity paradigm also encourages inclusion of neurodivergent people within research designs [29], yet only eight included studies openly acknowledged involving neurodivergent contributors. Author reflexivity [109] and consideration of the research team is essential, particularly due to known communication differences between neurotypical and neurodivergent people (the ‘*double empathy’* problem [110]). Neurotypical researchers should explicitly reflect on their position, so readers can consider this influence on study design and data interpretations, and so researchers can challenge their own perspectives. Researchers should also take steps to create research environments that can support the empowerment of neurodivergent participants. Co-production methodologies [111, 112] may be one way to achieve this. The Medical Schools Council emphasise the importance of actively including diverse student groups within medical education settings [113], but this is currently challenging for educators to achieve for neurodivergent students due to the lack of established literature in this area. The medical education community cannot expect the burden of this work to solely befall neurodivergent researchers, and neurotypical educators have a responsibility to use our privilege to enact change by elevating neurodivergent voices and removing disabling barriers.

### Limitations

The majority of authors of this review are neurotypical, which may influence the development of search terms and interpretation of studies. Neurotypical authors made efforts to improve their awareness of neurodivergent experiences through familiarization with neurodivergent-authored texts and discussion with neurodivergent colleagues, but we acknowledge this is not equivalent to lived experience. As discussed, the work to improve inclusivity of medical education for neurodivergent students should not solely fall on neurodivergent researchers, but authors should be explicit about their own experiences. The search strategy aimed to include a range of terms used to describe neurodivergence. However, the nature of a search strategy requires a finite list of terms, which may not be in keeping with the broad socio-political definition of neurodiversity, and relevant studies may have used terms not included in the search. A ‘NOT’ operator was required to produce manageable search results, and pragmatic factors reduced capacity for full independent screening of identified articles, heightening the risk of excluding potentially relevant studies. Backwards citation searching was employed to help mitigate this. The databases searched also skew heavily to the West, so alongside limitations introduced by only including studies in English, it is likely these findings are most suited to application in a Western context.

## Conclusion

There is a notable lack of empirical studies investigating educational practice for undergraduate neurodivergent students in medical education, with only 15 studies identified in this scoping review. Although the barriers facing neurodivergent students are clear, there is limited research regarding how educators can help remove these and improve support for this student group. Existing evidence suggests stigma and poor understanding by staff are present, meaning better training and awareness should be prioritized. Educators can adapt their practice, for example by relying less on traditional, didactic lectures, and being mindful of communication differences that may exist between neurodivergent students and staff, particularly during smaller group sessions. Consideration of UDL principles within teaching may be useful, but a more robust evidence base for this approach is necessary. These adaptations are likely to benefit all students, but will provide significant impact on enhancing inclusivity for those who are neurodivergent, which is vital to ensure our physician workforce is as diverse as the patient populations it serves. Future studies should further explore the learning experiences of students who are neurodivergent, for a range of conditions and for specific areas of the medical curriculum, to more clearly define their strengths and challenges, with the aim of developing clear, pragmatic, and evidence-based guidance for educators. Furthermore, research regarding assessment should extend to a variety of diagnoses beyond dyslexia and SpLDs, and interrogate our assessment approaches and standards themselves, not only the role of accommodations. In particular, assessment approaches and accommodations for practical clinical examinations should be further investigated. Explicit utilization of the neurodiversity paradigm is likely to be helpful in developing inclusive research practice, for example through use of co-production methods that can act to amplify neurodivergent voices. The limited evidence identified in this review indicates that there is a need for studies that can outline pragmatic changes for educators to implement in their practice, in order to celebrate and enhance the strengths that neurodivergent students bring to the medical profession.

## Supplementary Information

Additional file 1 – PRISMA-ScR Checklist.

Additional file 2 – Search Strategies.

Additional file 3 – Data Extraction Tool.

Additional file 4 – List of Included Studies.

## Data Availability

All data generated or analysed during this study are included in this published article and its supplementary information files.

## Abbreviations

HEIs: Higher Education Institutions
SpLD: Specific Learning Difficulty
ADHD: Attention-Deficit Hyperactivity Disorder
PRISMA-ScR: Preferred Reporting Items for Systematic Reviews and Meta Analyses for Scoping Reviews
OSF: Open Science Framework
OSCEs: Objective Structured Clinical Examinations
UDL: Universal Design for Learning
