# Supplementary material for: Undergraduate medical education for neurodivergent students: a scoping review
Source: BMC Med Educ. 2025 Dec 13;26:103. doi: 10.1186/s12909-025-08447-2 (PMC12822054; doi:10.1186/s12909-025-08447-2)
Supplement: Supplementary file 2 — Additional file 2 – Search Strategies. [file 12909_2025_8447_MOESM2_ESM.docx]

*Search Strategies*

| 1. **Portal/Interface:** Ovid |
| --- |
| **Database:** Medline - Ovid MEDLINE(R) and In-Process, In-Data-Review & Other Non-Indexed Citations 1946 to April 18, 2024 |
| **Date:** 19/04/2024 |
| **Limits:** None |
| **Search Terms:**   \| 1. \| exp Autism Spectrum Disorder/ \| \| --- \| --- \| \| 2. \| exp "Attention Deficit and Disruptive Behavior Disorders"/ \| \| 3. \| exp Learning Disabilities/ \| \| 4. \| neurodiver*.ti,ab,kf. \| \| 5. \| autis*.ti,ab,kf. \| \| 6. \| adhd.ti,ab,kf. \| \| 7. \| learning disab*.ti,ab,kf. \| \| 8. \| Intellectual disab*.ti,ab,kf. \| \| 9. \| learning difficult*.ti,ab,kf. \| \| 10. \| learning disorder*.ti,ab,kf. \| \| 11. \| learning disturbance*.ti,ab,kf. \| \| 12. \| learning impairment.ti,ab,kf. \| \| 13. \| learning problem*.ti,ab,kf. \| \| 14. \| attention deficit.ti,ab,kf. \| \| 15. \| attention disturbance*.ti,ab,kf. \| \| 16. \| dyslexi*.ti,ab,kf. \| \| 17. \| dyspraxi*.ti,ab,kf. \| \| 18. \| dyscalcu*.ti,ab,kf. \| \| 19. \| aphantasi*.ti,ab,kf. \| \| 20. \| autis* spectrum.ti,ab,kf. \| \| 21. \| or/1-20 \| \| 22. \| exp Education, Medical/ \| \| 23. \| exp Education, Nursing/ \| \| 24. \| exp Education, Dental/ \| \| 25. \| exp Education, Pharmacy/ \| \| 26. \| education, predental/ or education, premedical/ \| \| 27. \| exp Students, Health Occupations/ \| \| 28. \| Schools, Medical/ \| \| 29. \| Schools, Dental/ \| \| 30. \| Schools, Nursing/ \| \| 31. \| Schools, Pharmacy/ \| \| 32. \| ((medical or dental or nursing or pharmacy) adj6 education).ti,ab,kf. \| \| 33. \| ((medical or dental or nursing or pharmacy) adj6 student*).ti,ab,kf. \| \| 34. \| ((medical or dental or nursing or pharmacy) adj6 teach*).ti,ab,kf. \| \| 35. \| ((medical or dental or nursing or pharmacy) adj6 program*).ti,ab,kf. \| \| 36. \| ((medical or dental or nursing or pharmacy) adj6 curricul*).ti,ab,kf. \| \| 37. \| ((medical or dental or nursing or pharmacy) adj6 assess*).ti,ab,kf. \| \| 38. \| ((medical or dental or nursing or pharmacy) adj6 exam*).ti,ab,kf. \| \| 39. \| ((medical or dental or nursing or pharmacy) adj6 train*).ti,ab,kf. \| \| 40. \| ((medical or dental or nursing or pharmacy) adj6 study*).ti,ab,kf. \| \| 41. \| (anatomy adj6 (education or student* or teach* or program* or curricul* or assess* or exam* or train* or study*)).ti,ab,kf. \| \| 42. \| (physiology adj6 (education or student* or teach* or program* or curricul* or assess* or exam* or train* or study*)).ti,ab,kf. \| \| 43. \| (biomed* adj6 (education or student* or teach* or program* or curricul* or assess* or exam* or train* or study*)).ti,ab,kf. \| \| 44. \| (sport* adj6 (education or student* or teach* or program* or curricul* or assess* or exam* or train* or study*)).ti,ab,kf. \| \| 45. \| (nutrition adj6 (education or student* or teach* or program* or curricul* or assess* or exam* or train* or study*)).ti,ab,kf. \| \| 46. \| (speech adj6 (education or student* or teach* or program* or curricul* or assess* or exam* or train* or study*)).ti,ab,kf. \| \| 47. \| (healthcare professional adj6 (education or student* or teach* or program* or curricul* or assess* or exam* or train* or study*)).ti,ab,kf. \| \| 48. \| (medicine adj6 (education or student* or teach* or program* or curricul* or assess* or exam* or train* or study*)).ti,ab,kf. \| \| 49. \| (physician adj6 (education or student* or teach* or program* or curricul* or assess* or exam* or train* or study*)).ti,ab,kf. \| \| 50. \| or/22-49 \| \| 51. \| 21 and 50 \| \| 52. \| Child*.ti,ab,kf. \| \| 53. \| 51 not 52 \| |
| **Number of Results:** 1937 |

| **Portal/Interface:** Ovid |
| --- |
| **Database:** Embase 1974 to 2024 April 18 |
| **Date:** 19/04/2024 |
| **Limits:** None |
| **Search Terms:**   \| 1. \| exp Autism/ \| \| --- \| --- \| \| 2. \| exp attention deficit hyperactivity disorder/ \| \| 3. \| exp learning disorder/ \| \| 4. \| neurodiversity/ \| \| 5. \| intellectual impairment/ \| \| 6. \| neurodiver*.ti,ab,kf. \| \| 7. \| autis*.ti,ab,kf. \| \| 8. \| adhd.ti,ab,kf. \| \| 9. \| learning disab*.ti,ab,kf. \| \| 10. \| Intellectual disab*.ti,ab,kf. \| \| 11. \| learning difficult*.ti,ab,kf. \| \| 12. \| learning disorder*.ti,ab,kf. \| \| 13. \| learning disturbance*.ti,ab,kf. \| \| 14. \| learning impairment.ti,ab,kf. \| \| 15. \| learning problem*.ti,ab,kf. \| \| 16. \| attention deficit.ti,ab,kf. \| \| 17. \| attention disturbance*.ti,ab,kf. \| \| 18. \| dyslexi*.ti,ab,kf. \| \| 19. \| dyspraxi*.ti,ab,kf. \| \| 20. \| dyscalcu*.ti,ab,kf. \| \| 21. \| aphantasi*.ti,ab,kf. \| \| 22. \| autis* spectrum.ti,ab,kf. \| \| 23. \| or/1-22 \| \| 24. \| exp medical education/ \| \| 25. \| exp nursing education/ \| \| 26. \| exp dental education/ \| \| 27. \| exp pharmacy education/ \| \| 28. \| exp premedical student/ \| \| 29. \| exp health student/ \| \| 30. \| exp medical school/ \| \| 31. \| exp pharmacy school/ \| \| 32. \| ((medical or dental or predental or nursing or pharmacy) adj6 education).ti,ab,kf. \| \| 33. \| ((medical or dental or predental or nursing or pharmacy) adj6 student*).ti,ab,kf. \| \| 34. \| ((medical or dental or predental or nursing or pharmacy) adj6 teach*).ti,ab,kf. \| \| 35. \| ((medical or dental or predental or nursing or pharmacy) adj6 program*).ti,ab,kf. \| \| 36. \| ((medical or dental or predental or nursing or pharmacy) adj6 curricul*).ti,ab,kf. \| \| 37. \| ((medical or dental or predental or nursing or pharmacy) adj6 assess*).ti,ab,kf. \| \| 38. \| ((medical or dental or predental or nursing or pharmacy) adj6 exam*).ti,ab,kf. \| \| 39. \| ((medical or dental or predental or nursing or pharmacy) adj6 train*).ti,ab,kf. \| \| 40. \| ((medical or dental or predental or nursing or pharmacy) adj6 study*).ti,ab,kf. \| \| 41. \| (anatomy adj6 (education or student* or teach* or program* or curricul* or assess* or exam* or train* or study*)).ti,ab,kf. \| \| 42. \| (physiology adj6 (education or student* or teach* or program* or curricul* or assess* or exam* or train* or study*)).ti,ab,kf. \| \| 43. \| (biomed* adj6 (education or student* or teach* or program* or curricul* or assess* or exam* or train* or study*)).ti,ab,kf. \| \| 44. \| (sport* adj6 (education or student* or teach* or program* or curricul* or assess* or exam* or train* or study*)).ti,ab,kf. \| \| 45. \| (nutrition adj6 (education or student* or teach* or program* or curricul* or assess* or exam* or train* or study*)).ti,ab,kf. \| \| 46. \| (speech adj6 (education or student* or teach* or program* or curricul* or assess* or exam* or train* or study*)).ti,ab,kf. \| \| 47. \| (healthcare professional adj6 (education or student* or teach* or program* or curricul* or assess* or exam* or train* or study*)).ti,ab,kf. \| \| 48. \| (medicine adj6 (education or student* or teach* or program* or curricul* or assess* or exam* or train* or study*)).ti,ab,kf. \| \| 49. \| (physician adj6 (education or student* or teach* or program* or curricul* or assess* or exam* or train* or study*)).ti,ab,kf. \| \| 50. \| or/24-49 \| \| 51. \| 23 and 50 \| \| 52. \| Child*.ti,ab,kf. \| \| 53. \| 51 not 52 \| |
| **Number of Results:** 3894 |

| **Portal/Interface:** Ovid |
| --- |
| **Database:** APA PsycInfo 1806 to April Week 3 2024 |
| **Date:** 19/04/2024 |
| **Limits:** None |
| **Search Terms:**   \| 1. \| exp autism spectrum disorders/ \| \| --- \| --- \| \| 2. \| exp attention deficit disorder with hyperactivity/ \| \| 3. \| exp Learning Disabilities/ \| \| 4. \| exp neurodiversity/ \| \| 5. \| neurodiver*.ti,ab,id. \| \| 6. \| autis*.ti,ab,id. \| \| 7. \| adhd.ti,ab,id. \| \| 8. \| learning disab*.ti,ab,id. \| \| 9. \| Intellectual disab*.ti,ab,id. \| \| 10. \| learning difficult*.ti,ab,id. \| \| 11. \| learning disorder*.ti,ab,id. \| \| 12. \| learning disturbance*.ti,ab,id. \| \| 13. \| learning impairment.ti,ab,id. \| \| 14. \| learning problem*.ti,ab,id. \| \| 15. \| attention deficit.ti,ab,id. \| \| 16. \| attention disturbance*.ti,ab,id. \| \| 17. \| dyslexi*.ti,ab,id. \| \| 18. \| dyspraxi*.ti,ab,id. \| \| 19. \| dyscalcu*.ti,ab,id. \| \| 20. \| aphantasi*.ti,ab,id. \| \| 21. \| autis* spectrum.ti,ab,id. \| \| 22. \| or/1-21 \| \| 23. \| exp medical education/ \| \| 24. \| exp dental education/ \| \| 25. \| exp medical students/ \| \| 26. \| exp dental students/ \| \| 27. \| exp nursing students/ \| \| 28. \| ((medical or dental or nursing or pharmacy) adj6 school).ti,ab,id. \| \| 29. \| ((medical or dental or nursing or pharmacy) adj6 education).ti,ab,id. \| \| 30. \| ((medical or dental or nursing or pharmacy) adj6 student*).ti,ab,id. \| \| 31. \| ((medical or dental or nursing or pharmacy) adj6 teach*).ti,ab,id. \| \| 32. \| ((medical or dental or nursing or pharmacy) adj6 program*).ti,ab,id. \| \| 33. \| ((medical or dental or nursing or pharmacy) adj6 curricul*).ti,ab,id. \| \| 34. \| ((medical or dental or nursing or pharmacy) adj6 assess*).ti,ab,id. \| \| 35. \| ((medical or dental or nursing or pharmacy) adj6 exam*).ti,ab,id. \| \| 36. \| ((medical or dental or nursing or pharmacy) adj6 train*).ti,ab,id. \| \| 37. \| ((medical or dental or nursing or pharmacy) adj6 study*).ti,ab,id. \| \| 38. \| (anatomy adj6 (education or student* or teach* or program* or curricul* or assess* or exam* or train* or study*)).ti,ab,id. \| \| 39. \| (physiology adj6 (education or student* or teach* or program* or curricul* or assess* or exam* or train* or study*)).ti,ab,id. \| \| 40. \| (biomed* adj6 (education or student* or teach* or program* or curricul* or assess* or exam* or train* or study*)).ti,ab,id. \| \| 41. \| (sport* adj6 (education or student* or teach* or program* or curricul* or assess* or exam* or train* or study*)).ti,ab,id. \| \| 42. \| (nutrition adj6 (education or student* or teach* or program* or curricul* or assess* or exam* or train* or study*)).ti,ab,id. \| \| 43. \| (speech adj6 (education or student* or teach* or program* or curricul* or assess* or exam* or train* or study*)).ti,ab,id. \| \| 44. \| (healthcare professional adj6 (education or student* or teach* or program* or curricul* or assess* or exam* or train* or study*)).ti,ab,id. \| \| 45. \| (medicine adj6 (education or student* or teach* or program* or curricul* or assess* or exam* or train* or study*)).ti,ab,id. \| \| 46. \| (physician adj6 (education or student* or teach* or program* or curricul* or assess* or exam* or train* or study*)).ti,ab,id. \| \| 47. \| or/23-46 \| \| 48. \| 22 and 47 \| \| 49. \| Child*.ti,ab,id. \| \| 50. \| 48 not 49 \| |
| **Number of Results:** 1507 |

| **Portal/Interface:** EBSCOhost |
| --- |
| **Database:** ERIC |
| **Date:** 19/04/2024 |
| **Limits:** None |
| **Search Terms:**  neurodiver* OR autis* OR adhd OR “attention deficit” OR “learning disab*” OR “Intellectual disab*” OR “learning difficult*” OR “learning disorder*” OR “learning disturbance*” OR “learning impairment” OR “learning problem*” OR “attention deficit*” OR “attention disturbance*” OR dyslexi* OR dyspraxi* OR dyscalcu* OR aphantasi* OR “autis* spectrum”  AND  ((medical OR dental OR nursing OR pharmacy) N6 school) OR ((medical OR dental OR nursing OR pharmacy) N6 education) OR ((medical OR dental OR nursing OR pharmacy) N6 student*) OR ((medical OR dental OR nursing OR pharmacy) N6 teach*) OR ((medical OR dental OR nursing OR pharmacy) N6 program*) OR ((medical OR dental OR nursing OR pharmacy) N6 curricul*) OR ((medical OR dental OR nursing OR pharmacy) N6 assess*) OR ((medical OR dental OR nursing OR pharmacy) N6 exam*) OR ((medical OR dental OR nursing OR pharmacy) N6 train*) OR ((medical OR dental OR nursing OR pharmacy) N6 study*) OR (anatomy N6 (education OR student* OR teach* OR program* OR curricul* OR assess* OR exam* OR train* OR study*)) OR (physiology N6 (education OR student* OR teach* OR program* OR curricul* OR assess* OR exam* OR train* OR study*)) OR (biomed* N6 (education OR student* OR teach* OR program* OR curricul* OR assess* OR exam* OR train* OR study*)) OR (sport* N6 (education OR student* OR teach* OR program* OR curricul* OR assess* OR exam* OR train* OR study*)) OR (nutrition N6 (education OR student* OR teach* OR program* OR curricul* OR assess* OR exam* OR train* OR study*)) OR (speech N6 (education OR student* OR teach* OR program* OR curricul* OR assess* OR exam* OR train* OR study*)) OR (“healthcare professional” N6 (education OR student* OR teach* OR program* OR curricul* OR assess* OR exam* OR train* OR study*)) OR (medicine N6 (education OR student* OR teach* OR program* OR curricul* OR assess* OR exam* OR train* OR study*)) OR (physician N6 (education OR student* OR teach* OR program* OR curricul* OR assess* OR exam* OR train* OR study*))  NOT  Child* |
| **Number of Results:** 763 |

| **Portal/Interface:** ProQuest |
| --- |
| **Database:** Social Sciences Premium Collection |
| **Date:** 19/04/2024 |
| **Limits:** Fields – Anywhere except full text (NOFT) |
| **Search Terms:**  neurodiver* OR autis* OR adhd OR “attention deficit” OR “learning disab*” OR “Intellectual disab*” OR “learning difficult*” OR “learning disorder*” OR “learning disturbance*” OR “learning impairment” OR “learning problem*” OR “attention deficit*” OR “attention disturbance*” OR dyslexi* OR dyspraxi* OR dyscalcu* OR aphantasi* OR “autis* spectrum”  AND  ((medical OR dental OR nursing OR pharmacy) NEAR/6 school) OR ((medical OR dental OR nursing OR pharmacy) NEAR/6 education) OR ((medical OR dental OR nursing OR pharmacy) NEAR/6 student*) OR ((medical OR dental OR nursing OR pharmacy) NEAR/6 teach*) OR ((medical OR dental OR nursing OR pharmacy) NEAR/6 program*) OR ((medical OR dental OR nursing OR pharmacy) NEAR/6 curricul*) OR ((medical OR dental OR nursing OR pharmacy) NEAR/6 assess*) OR ((medical OR dental OR nursing OR pharmacy) NEAR/6 exam*) OR ((medical OR dental OR nursing OR pharmacy) NEAR/6 train*) OR ((medical OR dental OR nursing OR pharmacy) NEAR/6 study*) OR (anatomy NEAR/6 (education OR student* OR teach* OR program* OR curricul* OR assess* OR exam* OR train* OR study*)) OR (physiology NEAR/6 (education OR student* OR teach* OR program* OR curricul* OR assess* OR exam* OR train* OR study*)) OR (biomed* NEAR/6 (education OR student* OR teach* OR program* OR curricul* OR assess* OR exam* OR train* OR study*)) OR (sport* NEAR/6 (education OR student* OR teach* OR program* OR curricul* OR assess* OR exam* OR train* OR study*)) OR (nutrition NEAR/6 (education OR student* OR teach* OR program* OR curricul* OR assess* OR exam* OR train* OR study*)) OR (speech NEAR/6 (education OR student* OR teach* OR program* OR curricul* OR assess* OR exam* OR train* OR study*)) OR (“healthcare professional” NEAR/6 (education OR student* OR teach* OR program* OR curricul* OR assess* OR exam* OR train* OR study*)) OR (medicine NEAR/6 (education OR student* OR teach* OR program* OR curricul* OR assess* OR exam* OR train* OR study*)) OR (physician NEAR/6 (education OR student* OR teach* OR program* OR curricul* OR assess* OR exam* OR train* OR study*))  NOT  Child* |
| **Number of Results:** 2799 |

| **Portal/Interface:** Clarivate |
| --- |
| **Database:** Web of Science |
| **Date:** 19/04/2024 |
| **Limits:** None |
| **Search Terms:**  ((TS=(neurodiver* OR autis* OR adhd OR “attention deficit” OR “learning disab*” OR “Intellectual disab*” OR “learning difficult*” OR “learning disorder*” OR “learning disturbance*” OR “learning impairment” OR “learning problem*” OR “attention deficit*” OR “attention disturbance*” OR dyslexi* OR dyspraxi* OR dyscalcu* OR aphantasi* OR “autis* spectrum”)) AND TS=(((medical OR dental OR nursing OR pharmacy) NEAR/6 school) OR ((medical OR dental OR nursing OR pharmacy) NEAR/6 education) OR ((medical OR dental OR nursing OR pharmacy) NEAR/6 student*) OR ((medical OR dental OR nursing OR pharmacy) NEAR/6 teach*) OR ((medical OR dental OR nursing OR pharmacy) NEAR/6 program*) OR ((medical OR dental OR nursing OR pharmacy) NEAR/6 curricul*) OR ((medical OR dental OR nursing OR pharmacy) NEAR/6 assess*) OR ((medical OR dental OR nursing OR pharmacy) NEAR/6 exam*) OR ((medical OR dental OR nursing OR pharmacy) NEAR/6 train*) OR ((medical OR dental OR nursing OR pharmacy) NEAR/6 study*) OR (anatomy NEAR/6 (education OR student* OR teach* OR program* OR curricul* OR assess* OR exam* OR train* OR study*)) OR (physiology NEAR/6 (education OR student* OR teach* OR program* OR curricul* OR assess* OR exam* OR train* OR study*)) OR (biomed* NEAR/6 (education OR student* OR teach* OR program* OR curricul* OR assess* OR exam* OR train* OR study*)) OR (sport* NEAR/6 (education OR student* OR teach* OR program* OR curricul* OR assess* OR exam* OR train* OR study*)) OR (nutrition NEAR/6 (education OR student* OR teach* OR program* OR curricul* OR assess* OR exam* OR train* OR study*)) OR (speech NEAR/6 (education OR student* OR teach* OR program* OR curricul* OR assess* OR exam* OR train* OR study*)) OR (“healthcare professional” NEAR/6 (education OR student* OR teach* OR program* OR curricul* OR assess* OR exam* OR train* OR study*)) OR (medicine NEAR/6 (education OR student* OR teach* OR program* OR curricul* OR assess* OR exam* OR train* OR study*)) OR (physician NEAR/6 (education OR student* OR teach* OR program* OR curricul* OR assess* OR exam* OR train* OR study*)))) NOT TS=(Child*) |
| **Number of Results:** 2041 |
