# Supplementary material for: Undergraduate medical education for neurodivergent students: a scoping review
Source: BMC Med Educ. 2025 Dec 13;26:103. doi: 10.1186/s12909-025-08447-2 (PMC12822054; doi:10.1186/s12909-025-08447-2)
Supplement: Supplementary file 3 — Additional file 3 – Data Extraction Tool. [file 12909_2025_8447_MOESM3_ESM.docx]

*Data Extraction Tool*

| **Category** | **Entities Extracted** |
| --- | --- |
| Article Metadata | Author(s)  Year  Location  Journal  Title  DOI or URL |
| Methodology | Epistemology/Ontology  Data type (quantitative or qualitative)  Methodology and/or methods used  Theory or conceptual framework used |
| Participants | Degree/Course  Students or Staff?  Number  Nature of participant condition(s) (e.g. Autism, dyslexia)  Whether a formal diagnosis was required for participation Whether “Neurodiversity” was mentioned in the article |
| Context and Research Team | Which aspect(s) of education the study focuses on (e.g. assessment, student experience)  University or Clinical Placement based  Is the author(s) teaching staff, student, or other  Does the author(s) identify as neurodivergent/as having SpLD, or not, or not disclosed  Were neurodivergent/SpLD students/people involved in research team/design of study? |
| Objective and Outcomes | Study aim  Key results |
