# Supplementary material for: Undergraduate medical education for neurodivergent students: a scoping review
Source: BMC Med Educ. 2025 Dec 13;26:103. doi: 10.1186/s12909-025-08447-2 (PMC12822054; doi:10.1186/s12909-025-08447-2)
Supplement: Supplementary file 4 — Additional file 4 – List of Included Studies. [file 12909_2025_8447_MOESM4_ESM.docx]

*List of Included Studies*

Anderson JL, Shaw SCK. The Experiences of Medical Students and Junior Doctors with Dyslexia: A Survey Study. International Journal of Social Sciences & Educational Studies. 2020-11-26 2020;7(1):62-71. doi:doi:https://doi.org/10.23918/ijsses.v7i1p62

Bailey A, Grotowski M, Bailey S. Medical education: Accommodating students with ADHD. Medical teacher. 2023:1-6. doi:https://dx.doi.org/10.1080/0142159X.2023.2287984

Gibson S, Leinster S. How do students with dyslexia perform in extended matching questions, short answer questions and observed structured clinical examinations? Advances in health sciences education : theory and practice. 2011;16(3):395-404. doi:https://dx.doi.org/10.1007/s10459-011-9273-8

Godfrey-Harris M, Shaw SCK. The experiences of medical students with ADHD: A phenomenological study. PloS one. 2023;18(8):e0290513. doi:https://dx.doi.org/10.1371/journal.pone.0290513

Gray CP, Burr SA. Timing is key to providing modified assessments for students with specific learning difficulties. Perspectives on medical education. 2020;9(1):49-56. doi:https://dx.doi.org/10.1007/s40037-019-00553-4

Hennessy LR, Shaw SCK, Anderson JL. Medical Students’ Attitudes towards and Beliefs about Dyslexia: A Single-Centre Survey Study. International Journal of Social Sciences & Educational Studies. 2020;7(4):69-79. doi:https://doi.org/10.23918/ijsses.v7i4p69

Magnin E, Ryff I, Moulin T. Medical teachers' opinions about students with neurodevelopmental disorders and their management. BMC medical education. 2021;21(1):16. doi:https://dx.doi.org/10.1186/s12909-020-02413-w

McKendree J, Snowling MJ. Examination results of medical students with dyslexia. Medical Education. 2011;45(2):176-182. doi:10.1111/j.1365-2923.2010.03802.x

Ricketts C, Brice J, Coombes L. Are multiple choice tests fair to medical students with specific learning disabilities? Advances in health sciences education : theory and practice. 2010;15(2):265-75. doi:https://dx.doi.org/10.1007/s10459-009-9197-8

Rowlands A, Abbott S, Bevere G, Roberts CM. Medical students' perceptions and understanding of their specific learning difficulties. International Journal of Medical Education. 2013;4:200-206. doi:10.5116/ijme.524f.cd3f

Shaw SCK, Anderson JL, Grant AJ. Studying Medicine with Dyslexia: A Collaborative Autoethnography. The Qualitative Report. 2016;21(11):2036-2054.

Shaw SCK, Anderson JL. The experiences of medical students with dyslexia: An interpretive phenomenological study. Dyslexia (Chichester, England). 2018;24(3):220-233. doi:https://dx.doi.org/10.1002/dys.1587

Shaw SCK, Doherty M, Anderson JL. The experiences of autistic medical students: A phenomenological study. Medical education. 2023;57(10):971-979. doi:https://dx.doi.org/10.1111/medu.15119

Shaw SCK, Hennessy LR, Anderson JL. The learning experiences of dyslexic medical students during the COVID-19 pandemic: a phenomenological study. Advances in health sciences education : theory and practice. 2022;27(1):107-124. doi:https://dx.doi.org/10.1007/s10459-021-10074-7

Walker ER, Shaw SCK, Anderson JL. Dyspraxia in Medical Education: A Collaborative Autoethnography. The Qualitative Report. 2020;25(11):4072-4093.
